# Umbilical Cord Mesenchymal Stem Cell Secretome Improves Clinical Outcomes and Changes Biomarkers in Knee Osteoarthritis

**DOI:** 10.3390/jcm12227138

**Published:** 2023-11-16

**Authors:** Radiyati Umi Partan, Khoirun Mukhsinin Putra, Narisa Felinka Kusuma, Surya Darma, Muhammad Reagan, Putri Muthia, Afifah Salshabila Radiandina, MGS Irsan Saleh, Eddy Mart Salim

**Affiliations:** 1 Internal Medicine Department, Division of Rheumatology, Faculty of Medicine, Sriwijaya University—Dr. Mohammad Hoesin Hospital, Palembang 30126, Indonesia; 2 Stem Cell and Regenerative Therapies, From Bench to Market Program, Faculty of Life Science & Medicine, King’s College London, London WC2R 2LS, UK; 3 Department of Pharmacology, Faculty of Medicine, Sriwijaya University, Palembang 30126, Indonesia; irsan_saleh_hasani@yahoo.com; 4 Internal Medicine Department, Division of Allergy & Immunology, Faculty of Medicine, Sriwijaya University—Dr. Mohammad Hoesin Hospital, Palembang 30126, Indonesia

**Keywords:** umbilical cord mesenchymal stem cell (UC-MSC) secretome, hyaluronic acid, VAS, WOMAC, MMP-3, TGF-β1

## Abstract

(1) Background: The current treatment for osteoarthritis is ineffective due to its focus on pain relief and lack of cartilage repair. Viscosupplementation such as hyaluronic acid improves symptoms but remains unnoticed for several months. Researchers are exploring cell-based therapies such as mesenchymal stem cells secretome and mesenchymal stem cells, which can repair cartilage damage. The objective of the research is to evaluate and compare the effectiveness of the secretome derived from umbilical cord mesenchymal stem cells (UC-MSCs) with hyaluronic acid (HA). (2) Methods: An open-label clinical trial involving 30 knee osteoarthritis patients divided into two groups received UC-MSC secretome and hyaluronic acid doses. The study assessed clinical outcomes using VAS and WOMAC and measured MMP-3 and TGF-β1 levels before and after treatment. (3) Results: A study of 30 subjects found that the UC-MSC secretome group showed a decrease in pain in the OA knee compared to the HA group. The therapy was most effective after the third injection, and the group showed a decrease in the MMP-3 ratio and an increase in TGF-β1 compared to the hyaluronic acid group. (4) Conclusions: UC-MSC secretome intra-articular injections showed superior clinical improvement, biomarker changes, and no side effects compared to hyaluronic acid over a 5-week interval.

## 1. Introduction

Osteoarthritis (OA) is characterised by progressive damage to articular cartilage accompanied by subchondral bone thickening, osteophyte formation, and moderate chronic inflammation of the non-specific synovium. In OA, the inflammatory processes are local, chronic, mild, and predominantly mediated by the body’s natural immune system [1,2]. The prevalence of osteoarthritis worldwide is quite high and is among the most common musculoskeletal diseases [3]. According to the World Health Organisation (WHO), the worldwide incidence of OA for men over 60 is 9.6%, and for women it is 18%. In Indonesia, the prevalence of OA knee has reached 15.5% (39 million) for men and 12.7% (32 million) for women among a total population of 255 million [4].

OA is a condition that causes pain, resulting in an impairment in patients’ ability to take part in daily physical activities. One of the contributing factors to the symptoms of pain in knee OA is the presence of osteophytes, which are bony outgrowths, as well as the stretching of joint capsules resulting from an accumulation of fluid known as an effusion. The development of osteophytes and effusions is closely linked to joint inflammation. Proinflammatory cytokines cause the expression of catabolic enzymes, such as matrix metalloproteinases (MMPs), which results in the destruction of cartilage [5,6].

The main focus of OA treatment is not only to reduce pain but also to improve function and quality of life. Symptomatic treatment is the standard procedure for OA [7,8]. Analgesics with nonsteroidal anti-inflammatory drugs (NSAIDs) were administered to patients with OA to reduce pain symptoms. The pain related to OA is chronic and progressive, so the long-term effects of NSAID consumption are typically undesirable. The Arthritis, Rheumatism, and Ageing Medical Information System (ARAMIS) in the United States and Canada has obtained data indicating that patients with OA and rheumatoid arthritis are 2.5 to 5 times more likely than the general population to be treated for NSAIDs related gastrointestinal diseases, with 16,500 annual deaths related to NSAIDs [9]. Intra-articular injections of glucocorticoids and viscosupplements, such as hyaluronic acid, were one method for treating osteoarthritis. The effects of hyaluronic acid as a viscosupplement on symptom improvement were medium-term. It remains in the joints for several months. Additionally, this treatment yields inconsistent results [10,11].

The current treatment of OA is inadequate because it is only focused on reducing pain and has no effect on cartilage repair. Because of the limited number of effective treatments for OA, new strategies have to be developed. Numerous researchers and clinicians consider cell-based therapies such as mesenchymal stem cells (MSC) and mesenchymal stem cell (MSC) secretomes as potential treatments that can reduce inflammation and stimulate cartilage tissue regeneration. The secretome produced by mesenchyme stem cells is more stable than mesenchyme stem cells or stem cells themselves and can repair cartilage damage. It is also anti-inflammatory, immunomodulatory, angiogenesis-promoting, and anti-apoptotic [4,12,13].

For these reasons, this study aims to examine and compare the effectiveness of the secretome of umbilical cord mesenchymal stem cells (UC-MSC) and hyaluronic acid (HA). In our hypothesis, we propose that the secretome from umbilical cord mesenchymal stem cells (UC-MSCs) is more effective than hyaluronic acid (HA) in improving clinical manifestation and changing biomarkers in knee osteoarthritis.

## 2. Materials and Methods

The study had a randomised, open-label design with a 1:1 allocation ratio, and it fulfilled the specified inclusion criteria. The research was done at the Rheumatology Division of Dr. Mohammad Hoesin General Hospital in Palembang from July to December 2022. The study was conducted in accordance with the guidelines outlined in the Declaration of Helsinki by the researchers. The study (No. 58/kepkrsmh/2022) received permission from the Ethics Committee of Dr. Mohammad Hoesin General Hospital. The research protocol, identified as NCT05579665, was properly submitted to the Clinical Trials Registry.

### 2.1. Study Participants/Patient Selection

The study enrolled consecutive participants who were screened according to the knee osteoarthritis criteria established by the American College of Rheumatology (ACR) 1990. These patients provided their informed consent to participate in the trial after receiving a thorough explanation. The study included individuals of both genders, ranging from 30 to 60 years of age, who were diagnosed with grade 2 and 3 osteoarthritis (OA) according to the Kellgren and Lawrence (KL) scale, as determined by X-ray imaging. The exclusion criteria included severe pain with VAS > 7, knee effusion, glucocorticoid or other intra-articular injections within 3 months beforehand, stem cell or secretome therapy, active cardiac and pulmonary disorders, haematological problems, liver and renal disorders, as well as acute or chronic infection, other autoimmune disorders, rising CRP, LED > 40 mm/hour, or RF positivity. Furthermore, participants were eliminated from the trial if they reported experiencing escalating discomfort subsequent to the injection, which did not subside even after the administration of an analgesic (1000 mg of paracetamol).

### 2.2. Description of Umbilical Cord Mesenchymal Stem Cell (UC-MSC) Secretome and Hyaluronic Acid products

Each subject was administered either intra-articular UC-MSC or HA at weekly intervals for a duration of five weeks. Clinical experts administered the injections.

A stem cell’s secretome is a bioactive molecule or substance composed of soluble proteins, immune cells, growth factors, and nucleic acids. It has five active components, namely pro-collagen, keratinocyte growth factor, vascular endothelial growth factor, fibroblast growth factor, and stromal cell-derived factor. The secretome in this study is produced and packaged in a laboratory of Regenic PT. Bifarma Adiluhung, which has been certified as a good manufacturing practice by the Indonesian Food and Drug Authority (CN: PW S.01.04.1.3.333.09.21-0082) and has operational permission for processing stem cells from the Ministry of Health Indonesia (No: 11/1/10/KES/PMDN/2018), so the preparation is in sterile solution and free of antibiotics, preservatives, and red phenol. Each 2 mL tube contains 100 percent pure secretome and no extraneous synthetic serum or growth factor.

Hyaluronic acid is the prototypical member of a large family of saccharide biopolymers (glycosaminoglycans of mucopolysaccharides), which are essential components of all extracellular matrices, such as cartilage and synovial fluid. The hyaluronic acid used in this study was made by PT. Pratapa Nirmala (FAHRENHEIT) Indonesia under the trademark Umaron. Each 2 mL vial contains sodium hyaluronate.

### 2.3. Randomisation and Intervention

The participants who fulfilled the predetermined criteria for inclusion and exclusion were allocated into two groups using a randomisation procedure. The allocation of subjects into two groups was achieved using block randomisation in a 1:1 ratio, following the acquisition of their informed consent. The randomisation process was conducted using a random number generator accessible via the following link: https://stattrek.com/statistics/random-number-generator.aspx (accessed on 23 July 2022).

The first group was administered a 2 mL dose of UC-MSC Secretome, while the second group got the same amount of hyaluronic acid. The randomisation process was carried out by non-clinical staff.

### 2.4. Clinical Outcomes Measures

The pain levels of all participants were evaluated by the Visual Analogue Scale (VAS) and the Western Ontario and McMaster University Arthritis Index (WOMAC) questionnaires. A WOMAC questionnaire included 26 items designed to assess pain, two items aimed at evaluating stiffness, and 17 items focused on assessing functional limits and overall function in individuals diagnosed with knee osteoarthritis. VAS and WOMAC were assessed before receiving intraarticular injections. Subsequently, these assessments were conducted on a weekly basis for a duration of 5 weeks, followed by an additional evaluation period of 12 weeks. Clinical staff members who were not a part of the research team evaluated the VAS and WOMAC measures.

### 2.5. Biochemical Assay

The samples were subjected to examination at the Prodia Clinical Laboratory in Jakarta. A total volume of about 5 millilitres (mL) of blood was obtained from each participant both prior to and following the intervention, specifically at the 12-week mark. The enzyme-linked immunosorbent assay (ELISA) kit (Quantikine^®^ ELISA Human Total MMP-3, R&D Systems, Inc., Minneapolis, MN, USA, Cat: DMP300, Lot: P347150) was utilised to quantitatively evaluate the concentration of matrix metalloproteinase MMP-3. The measurement of Transforming Growth Factor Beta 1 (TGF-β1) levels was performed through the use of an enzyme-linked immunosorbent assay (ELISA) kit. Specifically, the Quantikine^®^ ELISA Human Total TGF-β1 kit, made by R&D Systems, Inc. in Minneapolis, MN, USA, was employed for this purpose. The examination utilised a particular kit, which was designated by the catalogue number DB100c and lot number P372273. The exams were carried out following the methodology outlined by the manufacturer of the equipment.

### 2.6. Statistical Analysis

The data analysis was performed by the SPSS 25.0 software programme (SPSS Corp., Chicago, IL, USA). The study provided information on the characteristics of the participants by presenting either a frequency or the mean value, together with the standard deviation for data that had a normal distribution. In cases where the data deviated from a normal distribution, the median value was employed in conjunction with the range spanning from the minimum through the maximum value. The objective of the study was to evaluate if it had a statistically significant disparity in the average (or median) results between the two groups, employing either the unpaired t-test or the Mann-Whitney U test. The scores of the same groups (pre-post) were assessed using either the paired t-test or the Wilcoxon test to see if there was a statistically significant improvement subsequent to the administration of supplements. A predetermined significance level of 0.05 or less was established to indicate statistical significance. The clinical and biochemical data were analysed and compared between the groups receiving UC-MSC secretome with hyaluronic acid at different time points. The primary research team carried out the analysis and interpretation of the findings.

## 3. Results

### 3.1. Baseline Characteristic

**Figure 1:**
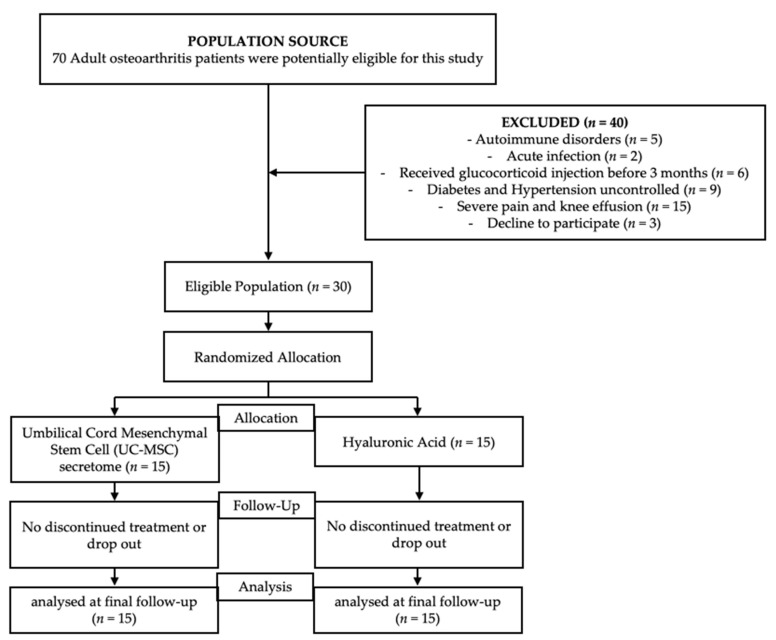
The Consolidated Standards of Reporting Trials (CONSORT) flowchart.

The study sample comprised 30 individuals who satisfied the predefined inclusion criteria at the beginning of the study. All participants in the study successfully completed the experimental task, as seen in Figure 1. Participants were recruited and thereafter monitored during the period of July to December 2022. The participants were randomly assigned and afterward divided into two groups: one group received the UC-MSC secretome (*n* = 15), whereas the other group received HA (*n* = 15).

Table 1 presents a brief overview of the basic characteristics identified in the patients. In this study, there were not any statistically significant differences found between the two groups across several demographic variables, including sex, body mass index (BMI), physical activity objectives, Kellgren Lawrence grade for osteoarthritis (OA), or concentrations of 25(OH)D. The majority of the participants in the study were predominantly women, with osteoarthritis classified as grade Kellgren Lawrence 2–3. Additionally, they reported participating in low levels of physical activity and had insufficient levels of serum 25(OH)D.

### 3.2. Clinical Results

The clinical evaluation of UC-MSC secretome and HA results after treatment included the assessment of patients through subjective and objective evaluations. These assessments were conducted at multiple time points, including the initial evaluation, weeks 1–5 of treatment, and the week following the completion of the 12-week treatment period.

**Figure 2:**
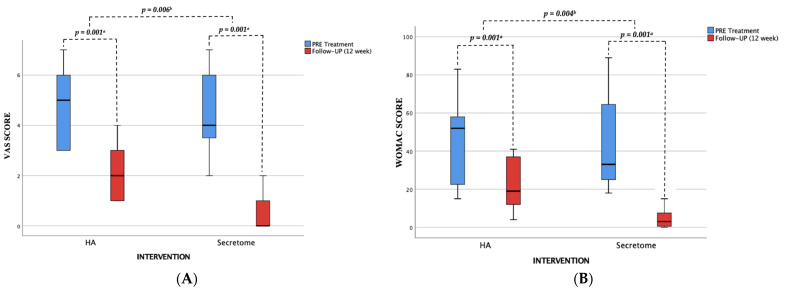
Box-and-whisker plot displaying the treatment effect of UC-MSC secretome and hyaluronic acid (HA) pre- and post-treatment measurement for week 12. The clinical outcomes of the therapy will be assessed based on two established measurement tools: (**A**) A Visual Analogue Scale (VAS) and (**B**) A Western Ontario and McMaster University Osteoarthritis Index (WOMAC). ^a^ Wilcoxon Test; ^b^ Mann–Whitney Test, the statistically significant difference before and after evaluations (*p* < 0.05).

This study aimed to assess the clinical outcomes of this intervention. Based on the assessment of pain levels using a visual analogue scale (VAS) and a Western Ontario and McMaster Universities Osteoarthritis Index (WOMAC) (see Figure 2), it was observed that both the UC-MSC Secretome group and the Hyaluronic Acid group showed a reduction in their mean pain scores over a 12-week duration. In the UC-MCS Secretome Group, the knee OA score decreased significantly, as measured by VAS −4 (95% CI, mean ± SD −4.06 ± 1.48) and WOMAC −41 (95% CI, mean ± SD −41.06 ± 24.02). This reduction was identified to be greater than the decrease observed in the hyaluronic acid group, which achieved an average pain reduction shown by VAS −3 (95% CI, mean ± SD 2.60 ± 1.24) and a mean decrease in WOMAC −21 (95% CI, mean ± SD −21.13 ± 14.07). The statistical analysis revealed a significant difference in the mean decrease of pain scores (measured by VAS and WOMAC) before and after treatment between the UC-MSC Secretome and hyaluronic acid (*p* < 0.05).

**Figure 3:**
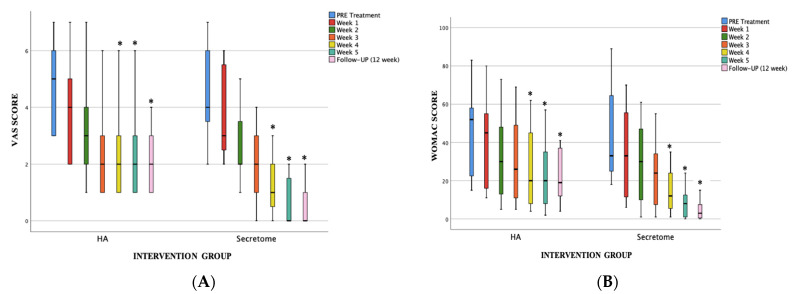
A box-and-whisker plot is used to visually represent the therapeutic effect of UC-MSC secretome and hyaluronic acid over a period of time. Evaluate the treatment’s clinical results according to the (**A**) Visual Analogue Scale (VAS) and (**B**) Western Ontario and McMaster University Osteoarthritis Index (WOMAC). * significant in statistical terms (*p* < 0.05) at an identifiable point.

A weekly assessment is conducted to identify the optimal time for measuring pain intensity using VAS and WOMAC indicators (see Figure 3). According to the results, the administration of the UC-MSC secretome for the third injection demonstrates a statistically significant decrease in VAS and WOMAC assessments (*p* < 0.05).

### 3.3. Biochemical Results

**Figure 4:**
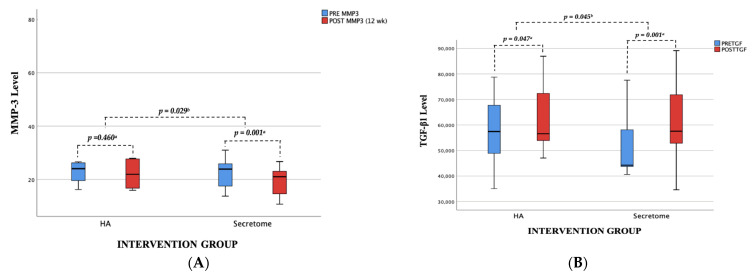
The biochemical findings pre- and post-therapy were compared between UC-MSC secretome and HA. The study examined the mean blood levels of (**A**) MMP-3 and (**B**) TGF-β1 before and 12 weeks after therapy evaluation. MMP-3: Matrix Metalloproteinase 3, TGF-β1: Transforming Growth Factor Beta 1, ^a^ Wilcoxon Test; ^b^ Mann–Whitney Test, statistically significant difference before and after evaluations (*p* < 0.05).

Following a period of 12 weeks of monitoring, it was found that the UC-MSC secretome and hyaluronic acid groups displayed a decrease in the MMP-3 ratio and an increase in TGF-β1 concentrations. In the UC-MSc Secretome group, the MMP3 ratio revealed a reduction of −2.81 ng/mL (95% CI, mean ± SD −2.82 ± 1.50), while the TGF-β1 levels revealed an increase of 10,221.60 pg/mL (95% CI, mean ± SD 10,221, 60 ± 9560.56), which was shown to be higher compared to the hyaluronic acid group. The statistical analysis of the data (Figure 4) revealed a significant difference in the rate of MMP-3 reduction and TGF-β1 increase before and after the intervention between UC-MSC secretome and hyaluronic acid (*p* < 0.05).

## 4. Discussion

The symptoms of pain in the knee resulting from OA often motivate individuals to look into health care. The severity of pain might demonstrate variability dependent on the level of joint impairment and differs among individuals and joint types. The main objective of an intraarticular injection is the alleviation of pain. To our knowledge, this study is the first clinical trial that has evaluated the comparative effects of administering UC-MSC secretome and HA in two different patient groups, assessing both subjective outcomes and biomarkers.

Both groups demonstrated statistically significant improvements in VAS and WOMAC scores, starting one week following the initial injection and continuing up to 12 weeks after the injection. Based on the findings, the administration of UC-MSC secretome for the third time leads to a statistically significant reduction in VAS and WOMAC scores. In the UC-MSC Secretome group, there was a significant decrease in mean VAS ratings from 5 to 1, representing an 80% reduction from the baseline after a 12-week period. In contrast, the hyaluronic acid group reported a VAS pain reduction from 5 to 2, equivalent to a 60% decrease. Both groups demonstrated significant improvements in WOMAC scores throughout the 12-week period. The mean WOMAC scores in the UC-MSC Secretome group showed a significant decrease from 46 to 5, representing an 89% reduction from the baseline after a 12-week period. In contrast, the hyaluronic acid group observed a decrease from 45 to 21, which equates to a reduction of around 47%. The achievement of this level of pain reduction indicates a successful outcome in clinical studies related to chronic pain, according to the established standards, which consider a decrease of at least 30% as indicative of considerable clinically meaningful differences [14].

Numerous clinical investigations have been undertaken to examine the effectiveness of therapy using the UC-MSC secretome in individuals diagnosed with osteoarthritis (OA). In research undertaken by Dilogo et al., a Phase I/II clinical trial was conducted, including a group of 29 patients with knee OA. The findings of the study demonstrated a statistically significant decrease in VAS and WOMAC scores among patients with knee OA [15]. In an animal model of OA, Khatab et al. found that the secretome of mesenchymal stem cells could relieve pain and protect cartilage [16]. In another study, Matas et al. gave 26 people injections of secretome of mesenchymal stem cells into their knee joints. The study findings demonstrated a significant improvement in both pain reduction and functional improvement, as seen by lower ratings on the WOMAC and VAS pain scales [17,18].

Furthermore, both groups reveal changes in biochemical testing that accompany their clinical benefits. The results of this study suggest that the group that received intraarticular injections of UC-MSC secretome exhibited a statistically significant reduction in MMP-3 levels and an increase in TGF-β1 levels at the 12-week after injection, in contrast to the group that received hyaluronic acid. The investigation of the UC-MSC secretome for potential therapeutic applications in OA has been the subject of in vitro studies. Saulnier et al. conducted a study whereby the secretome of UC-MSCs was employed for the treatment of osteoarthritis (OA) in rabbit IL-1β synoviocytes, which were specifically chosen as the target cells. The findings of the study demonstrated a reduction in the levels of MMP-1, MMP-3, MMP-13, IL-1β, and TIMP. These observed changes have the potential to prevent the progression of the OA process. Widowati et al. conducted a study to examine the impact of interleukin-1β (IL-1β) targeting on a human chondrocyte cell line. The findings exhibited a rise in insulin-like growth factor-1 (IGF-1), a factor linked to the process of chondrogenesis. Furthermore, the research revealed a notable reduction in the expression of ADAMTS1, MMP-1, and MMP-3, which play a crucial role in the degenerative mechanisms associated with osteoarthritis [4].

Prostaglandins, tumour necrosis factor (TNF-α), interleukin (IL)-1, IL-6, and nitric oxide are inflammatory cytokines that have been associated with the advancement of osteoarthritis (OA). In vitro models have also identified these variables as highly effective stimulators of cartilage injury, achieved by activating the NF-kB pathway and inducing the formation of cyclooxygenase-2 (COX-2). Several investigations have demonstrated that the reduction of pain in osteoarthritis (OA) can be related to the downregulation of joint inflammation. This effect is considered to result from the inhibition of nuclear kappa factor B (NF-κB) and cyclooxygenase-2 (COX-2), both of which are essential components in the inflammatory pathway and have a direct impact on the activation of osteoblast progenitor cells and chondrocytes [18,19].

**Figure 5:**
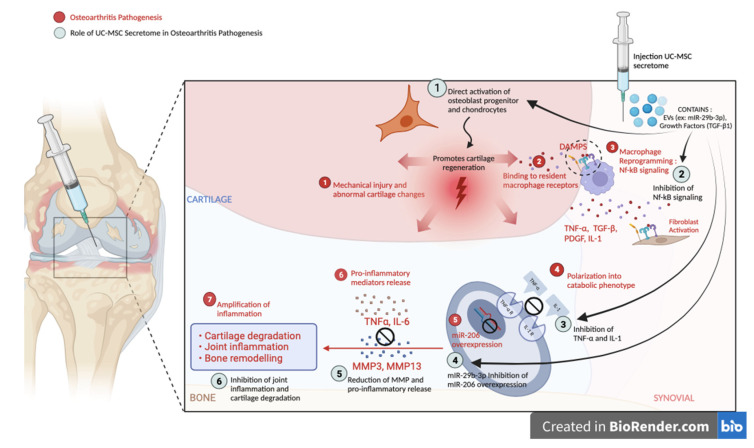
The Effect of the UC-MSC Secretome in the Pathogenesis of Osteoarthritis. Evs: Extracellular vesicles, IL: interleukin, NF-kB: Nuklear factor kappa B, MMP: Matrix Metalloproteinase, miR: MicroRNA. (The figure was created and generated on the online platform BioRender.com—access date: 11 September 2023).

In Figure 5 illustrates the crucial role of the UC-MSC secretome in the progression of osteoarthritis (OA). The UC-MSC secretome has been seen to have a beneficial impact on the structure of cartilage. This effect can be attributed to the presence of several growth hormones (TGF-β1) that possess the ability to directly stimulate the proliferation of chondrocytes, thereby promoting cartilage regeneration. Additionally, TGF-β1 signaling is involved in protecting cartilage destruction by counteracting the catabolic effects of pro-inflammatory cytokines. Particularly, such growth-promoting properties are not observed in the hyaluronic acid group [20].

The secretome is known to contain additional vesicles called extracellular vesicles (EVs) that contain RNA KLF3-AS1 and miR-29b-3p [21,22]. This RNA has the ability to inhibit the activity of miR-206, which has been associated with the progression of OA and the promotion of senescence, a characteristic frequently observed in OA. Furthermore, these EVs also contain miR-21, which exhibits anti-inflammatory activity by downregulating TNF-α [23,24,25].

The secretomes of MSCs were observed to exhibit distinct characteristics compared to the secretomes of other kinds of cells. The observed differences included the presence of angiogenic factors, reduced levels of metalloproteinases (MMPs), and elevated synthesis of TGF-β1, chemokines, and anti-inflammatory cytokines. These characteristics suggest that the UC-MSC Secretome shows the ability to control inflammatory responses [23,24]. The administration of secretome injections in models of OA has been found to trigger an increase in the levels of TGF-β1, aggrecan, SOX-9, and collagen type II. These molecular markers are recognised as key components of the pathways that regulate cartilage repair and regeneration [26,27].

In summary, our study, in addition to supporting previous studies, demonstrates that injections of UC-MSC secretome have the potential to provide persistent discomfort alleviation, functional benefits, and potential cartilage regeneration in the knee joint. HA may offer certain immediate benefits in both pain alleviation and enhanced functionality. However, repeated administration of the UC-MSC secretome demonstrates a potential superiority since it has the ability to possibly change the progression of the disease. Further investigation is required; nevertheless, the UC-MSC secretome has promise as a regenerative therapy for knee osteoarthritis. During the 12-week administration of UC-MSC Secretome, there were no adverse effects observed in this study.

The study has many limitations. The investigation was conducted in a single research facility and was confined to individuals diagnosed with grade 2–3 knee osteoarthritis, as determined by the Kellgren and Lawrence classification criteria. The findings lacked generalisability across different osteoarthritis groups exhibiting diverse levels of radiographic severity. Furthermore, it should be noted that the sample size utilised in the study was rather small, and the duration of the follow-up period was relatively brief. To investigate long-term efficacy, it is recommended to conduct larger studies with extended follow-up. Lastly, we did not directly evaluate cartilage using high-resolution magnetic resonance imaging or histological analysis, which could have provided more objective data for the evaluation of efficacy.

## 5. Conclusions

This study presented results that the administration of UC-MSC secretome and hyaluronic acid effectively influenced the reduction of pain based on VAS and WOMAC indicators in knee osteoarthritis patients, with a more significant decrease in pain in the UC-MSC secretome compared to the hyaluronic acid. Achieving a significant difference in subjective outcomes favouring the UC-MSC secretome, there was a trend towards a decrease in MMP-3 and an increase in TGF-β1 in the biochemical measurements. We suggest conducting additional high-quality studies that would support the results.

## Figures and Tables

**Table 1:** Baseline characteristics before treatment.

Variable	Total *n*:30 (%)	Intervention Group		*p*
		UC-MSC Secretome (%)	HA (%)	
Gender				0.100 ^a^
Female	28 (93)	14 (93)	14 (93)	
Male	2 (7)	1 (7)	1 (7)	
BMI				
Severe Underweight (<17)				0.056 ^a^
Underweight (17–18.4)				
Normal (18.5–25)	6 (20)	3 (20)	3 (30)	
Overweight (25.1–27)	12 (30)	6 (30)	6 (30)	
Obese (>27)	12 (30)	6 (30)	6 (30)	
Physical Activity				0.232 ^a^
Mild	9 (30)	3 (20)	6 (40)	
Moderate	21 (70)	12 (80)	9 (60)	
Severe				
Kellgren Lawrence Grade				0.068 ^a^
Grade 2	15 (50)	10 (67)	5 (33)	
Grade 3	15 (50)	5 (33)	10 (67)	
Serum 25(OH)D Level				0.057 ^a^
Normal				
Insufficiency	20 (67)	10 (67)	10 (67)	
Deficiency	10 (33)	5 (33)	5 (33)	

^a:^ Chi-Square Test.

## Data Availability

The author of this study will disclose the raw data supporting the conclusion without any unjustified concerns.
